# Prevalence and prevention of brucellosis in cattle in Lebanon

**DOI:** 10.14202/vetworld.2020.364-371

**Published:** 2020-02-27

**Authors:** Hussein Hassan, Ali Salami, Nada Nehme, Raed Al Hakeem, Jeanne El Hage, Rana Awada

**Affiliations:** 1 Department of Natural Sciences, School of Arts and Sciences, Lebanese American University, Lebanon; 2 Rammal Hassan Rammal Research Laboratory, Physio-toxicity (PhyTox) Research Group, Faculty of Sciences (V), Lebanese University, Nabatieh, Lebanon; 3 Department of Veterinary Sciences, Faculty of Agronomy, Lebanese University, Dekwaneh, Lebanon; 4 Animal Health Laboratory, Lebanese Agricultural Research Institute, Fanar, Lebanon

**Keywords:** *Brucella abortus* and *melitensis*, brucellosis, public health, vaccines, zoonosis

## Abstract

**Background and Aim:**

Brucellosis is a zoonotic disease caused by the bacterium of the genus *Brucella*. This disease is present worldwide, especially in developing and underdeveloped countries, where it is endemic. This first-of-its-kind study in Lebanon aimed to assess the prevalence of brucellosis across the country and to determine the efficacy of a vaccine for reducing losses in herds so that its toll on public health is reduced.

**Materials and Methods:**

Three hundred and fifty-three blood serum and 261 milk samples were obtained from cows in different areas of Lebanon. The samples were analyzed using serological tests (rose Bengal, milk ring, and indirect enzyme-linked immunosorbent assay [ELISA]) and confirmed with competitive ELISA and polymerase chain reaction.

**Results:**

The highest rate of *Brucellae* was found in the Bekaa region (10%). After vaccination of 5 cows and 13 heifers at different times, the results showed that all the vaccinated animals have developed an immune response to brucellosis 60 days after vaccination. This vaccine can be considered as stable and preventative to protect against brucellosis in animals and thus protect the public from this infection.

**Conclusion:**

These findings will provide further insight into designing future targeted awareness interventions and adapted policies as efforts toward reducing the prevalence and prevention of brucellosis in cattle in Lebanon.

## Introduction

One of the most important zoonotic foodborne diseases in the world, brucellosis, is still present in Lebanon and is causing much distress to the country [1]. It goes back the 79 AD when it first appeared and mutated along the years. Since the 1800s, studies have more accurately isolated this bacterium and its different strains and their subtypes/biotypes, mainly by Dr. Bruce who differentiated between brucellosis and typhoid, hence giving it its name [2]. Brucellosis is caused by *Brucella* spp. They are Gram-negative, non-encapsulated, non-sporulated, and non-motile coccobacilli. They belong to *Alphaproteobacteria* and have progressed with years, reaching various animal kingdoms of the world, including humans. *Brucella* infects different species and can be transmitted within and between the different animal species. At present, there are 12 species of *Brucella*, whereby four of them affect humans: *Brucella melitensis*, *Brucella abortus*, *Brucella suis*, and *Brucella canis*. Regarding the cattle, *B. abortus* is the main disease inducers. *B. melitensis* has the most pathogenicity for humans [3].

The main mode of transmission of *Brucella* bacterium in humans is through the ingestion of contaminated dairy products. Here comes the necessity of the pasteurization of milk. Other modes of transmission are direct contact with the infected animal and its excreta or in minor possibility through aerosols. On the other hand, the animal gets infected through contaminated food, water, fomites, contact with an infected animal, abortion and mating, as well as other routes such as artificial insemination using semen from infected males. The most observed sign of brucellosis is abortion in ruminants including cows, sheep, and goats; other signs include stillbirth, retained placenta, and decrease in milk production. However, in bulls, orchitis and epididymitis are the main clinical signs. For humans, symptoms include undulant fever, joint pain, headache, respiratory distress, and septicemia [4]. It is widespread and found in most regions of the developing countries such as Latin America, North and East Africa, and South and Central Asia including the Middle East [5]. In the Arab World, Syria has the highest human brucellosis incidence over the years followed by Iraq, Iran, and Saudi Arabia ranking world widely as 4^th^, 6^th^, and 7^th^, respectively [6].

Various developed countries had eradication or control programs to reduce the incidence of infection by *Brucella* spp., for example, West and North Europe, Canada, Japan, Australia, and New Zealand, and are considered now free from *Brucella* infection [7]. In Lebanon, it was noticed that the increase of the cases of brucellosis was in the southern and northern areas. In addition, the rural regions showed the highest rate of infection due to the livestock holders living in proximity to the farms, coming in contact with the animals as well as consuming raw milk and milk products [8]. It is still to this day 1 of the ongoing bacterial foodborne diseases in Lebanon [9].

One of the most effective control methods for this disease was the vaccination and eradication/quarantine of the infected animals. However, there are no vaccines for humans; hence, this emphasizes the need to control this disease in animals and humans [10].

This study aimed to determine the prevalence of brucellosis in Lebanon and propose preventative measures through the usage of a vaccine.

## Materials and Methods

### Ethical approval

Ethical approval was not needed for this study. A licensed veterinarian was present throughout this study with respect to animal welfare. No animal was harmed during the sample collection.

### Samples collection

Three hundred and fifty-three blood samples and 261 milk of cattle in different ages were collected from 50 farms in several regions of Bekaa (Baalbek, Zahle, Yunin, Hermel, Shaat, Hadad, and Harbata) and Mount Lebanon (Saoufar, Beit Chabeb, Kfardebian, Hrajel, Bkaatouta, Jbeil, Amchit, and Mayfouk). Cattle were mostly 2-5 years old. The cattle over 7 years were generally unguarded, and those under 2 years were not yet accustomed to contact with humans, and breeders were afraid that the blood sample would stress the little ones. Milk samples obtained from the animals were kept refrigerated at 4°C overnight before examination by milk ring test (MRT), while the blood samples collected were centrifuged and their serums were stored at −20°C before be tested by rose Bengal test (RBT) and enzyme-linked immunosorbent assay (ELISA), indirect and competitive.

### Serological test

#### MRT

One milliliter of milk was placed in Eppendorf tubes and 30 µl of *B. abortus* antigen was added (standardized *B. abortus*, MRT antigen – AHVLA – UK). The milk-antigen mixture was then incubated at 37°C for 1 h.

#### RBT

Thirty microliters of rose Bengal solution (Pourquier, rose Bengal antigen – IDEXX – Montpellier – France) were added to 50 µl of serum on a white glossy ceramic tile. The tile was then rocked at room temperature for 3 min. Any granulation formation was considered positive.

#### Indirect ELISA

Four hundred and fifty milliliters of distilled water and 50 mL of “wash” solution were used. As it is a quick or short incubation, 90 µL of the diluted “wash” solution, taken using a multipipette, was placed in the wells. Then, 10 µL of undiluted serum was taken with a micropipette, and 10 µL positive and negative controls, to reach a total dilution of 1:10. The wells were shaken gently, covered, and incubated at 37°C in a humid incubator for 60 min. Afterward, the wells were washed with 300 µL of the “wash” solution diluted 3 times. After the last wash, the water was removed by gently tapping the wells on absorbent paper. Then, 100 µL of the conjugate was added to each well and incubated again at 37°C and wet for 60 min. Again, washes were repeated, and 100 µL of TBM # 12 or # 12 were added, the wells were shaken, covered, and incubated at 18-26°C for 15 min. Finally, the reaction was stopped by adding 100 µL of the “stop” solution No. 3 to each well and the results were read using a photometer with a wavelength of 450 nm.

#### Competitive ELISA

Brucella-Ab C-ELISA kit was used on selected positive and negative samples for further confirmation. It is highly specific and highly discriminative between antibodies from vaccination (strain 19) and those from infection according to the manufacturer SVANOVIR^®^. First, the “Sample Dilution Buffer” solution was put in all the wells. Five microliters of the positive control (PC), low positive (FP), and negative (NC) were added in duplicate. “Sample Dilution Buffer” was added in the appropriate wells and designated in “Conjugate Control” (CC), and same, serum of the samples was added, in duplicate. Then, mAb solution was added, and wells were covered, shaken well, and incubated at room temperature for ½ h. Then, the wells were washed and taped with the PBS-Tween buffer solution. The conjugate solution was added and incubated at room temperature for ½ h. The washing step was repeated, “Substrate solution” was added, incubated for 10 min, and then stopped.

### Preventative measures through vaccine

To study the effectiveness of the vaccine, the licensed vaccine was administered to two groups; five mature lactating cows as the first group and 13 heifers as the second group. The vaccine was given subcutaneously in the elbow region with a dosage of 3 mL of reconstituted Brucevac^®^ (full dose) (0.5-4.0 × 10^9^ CFU) freeze-dried live attenuated *B. melitensis* strain Rev.1. The timeline used to observe and evaluate the development of the vaccine was as follows: Days 0, 7, 14, 21, 30, and 60; knowing that on day 21, there was dissemination in blood, and on day 60, there was shedding in milk. The tests used for confirmation were RBT, indirect ELISA (iELISA), and polymerase chain reaction (PCR).

### PCR

PCR was used in this study to follow-up on the progression of the pathogen in the vaccinated animals, whether animals are shedding the attenuated form of the live pathogen in milk or whether or not there is dissemination in blood and the time at which dissemination and shedding would stop. For PCR, we used the protocol of blood DNA extraction (Norgen Biotek Corporation, 2018) recommended by the manufacturer.

Before the detection of *Brucella-* specific components, it was necessary to amplify the mixtures *VetPCR^™^* *B. melitensis* premixture with DNase/RNase-free water. *B. melitensis* PCR-positive or negative control was added. All samples were placed in a thermocycler to perform amplification. First cycle in the amplification program was the initial denaturation phase that was conducted at a temperature of 94°C for 2 min followed by 30 cycles that consisted of three phases: Denaturation for 30 s at 94°C, annealing next for 30 s at 58°C, and finally extension for 30 s at 72°C for each cycle. The final cycle consisted of the final extension and took 5 min at 72°C. The final step was the detection of amplified products by agarose gel electrophoresis on an ultraviolet transilluminator.

### Statistical analysis

Statistical analyses were performed using SPSS (SPSS Statistics for Windows Version 22.0, IBM, Armonk, NY). This software was used as well for data management and cleaning. Descriptive statistics were carried out and reported as frequencies and percentages for categorical variables. The Chi-square test was used to assess any significant difference between the categorical variables. The level of significance was set at p<0.05 for all statistical analyses. The iELISA used in this study allows for qualitative evaluation of the quality of humoral immunity (antibodies Ig). The results were read as S/P ratio, and results under 110% were judged negative; whereas results strictly over 120% were considered positive. Between 110% and 120% were considered inconclusive or insufficient to judge a sample positive.

## Results

### Bovine brucellosis rate by MRT test according to age, gender, and regions

Table-1 summarizes the bovine brucellosis rate using MRT test according to age, gender, and regions. Thirty-three cases among 261 tested positives. All were females (33 cases) and the majority were aged between 2 and 5 years (32 cases). Table-1 shows that bovine brucellosis was significantly higher in Bekaa than in Mount Lebanon (p<0.001). Results also showed a significant association between age and MRT test results (p<0.001).

**Table-1:** Bovine brucellosis rate using MRT test according to age, gender, and regions.

Variables	MRT						

	Negative (%)	Positive (%)	Invalid (%)	Pregnant (%)	Inconclusive (%)	Total	p-value
Region, n (%)							<0.001
Bekaa	97 (48.3)	25 (12.4)	55 (27.4)	10 (5.0)	14 (7.0)	201	
Mount Lebanon	107 (70.4)	8 (5.3)	37 (24.3)	0 (0.0)	0 (0.0)	152	
Gender, n (%)							<0.001
Female	204 (59.1)	33 (9.6)	84 (24.3)	10 (2.9)	14 (4.1)	345	
Male	0 (0.0)	0 (0.0)	8 (100)	0 (0.0)	0 (0.0)	8	
Age, n (%)							<0.001
<2 years	0 (0.0)	0 (0.0)	27 (100)	0 (0.0)	0 (0.0)	27	
2-5 years	177 (60.6)	32 (11.0)	61 (20.9)	8 (2.7)	14 (4.8)	292	
>5 years	26 (76.5)	1 (2.9)	5 (14.7)	2 (5.9)	0 (0.0)	34	

MRT: Milk ring test

### Bovine brucellosis rate by RBT and B. abortus iELISA tests according to age, gender, and regions

Table-2 summarizes the bovine brucellosis rate using RBT and *B. abortus* indirect ELISA tests according to age, gender, and regions. Twenty-eight cases among 353 tested positive for RBT and 37 cases among 353 for *B. abortus* indirect ELISA test. For the two tests, all positive cases were females between 2 and 5 years old. Table-2 shows, for the two tests, that bovine brucellosis was higher significantly in Bekaa than in Mount Lebanon (p<0.001). Results also showed a significant association between age and test results (p<0.001).

**Table-2:** Bovine brucellosis rate using RBT and *B. abortus* indirect ELISA tests according to age, gender, and regions.

Variables	RB				ELISA antibody			
	
	Negative (%)	Positive (%)	Total	p-value	Negative (%)	Positive (%)	Total	p-value
Region, n (%)				0.016				0.015
Bekaa	179 (89.1)	22 (10.9)	201		173 (86.1)	28 (13.9)	201	
Mount Lebanon	146 (96.1)	6 (3.9)	152		143 (94.1)	9 (5.9)	152	
Gender, n (%)				0.401				0.328
Female	317 (91.9)	28 (8.1)	345		308 (89.3)	37 (10.7)	345	
Male	8 (100)	0 (0.0)	8		8 (100)	0 (0.0)	8	
Age, n (%)				0.042				0.013
<2 years	27 (100)	0 (0.0)	27		27 (100)	0 (0.0)	27	
2-5 years	264 (90.4)	28 (9.6)	292		255 (87.3)	37 (12.7)	292	
>5 years	34 (100)	0 (0.0)	34		34 (100)	0 (0.0)	34	

RB=Retinoblastoma, ELISA=Enzyme-linked immunosorbent assay

### Bovine brucellosis rate by MRT test

**Figure-1:**
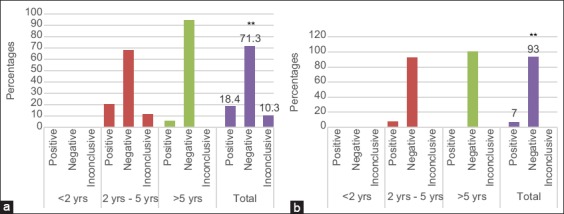
Variation of Bovine brucellosis rate by MRT with age in the two selected regions: Bekaa (a) Mount lebanon (b).

The MRT test results showed that the rate of brucellosis in cattle varied with age in the two selected regions. In Bekaa, 20% of the samples taken from cattle aged 2-5 years were positive, 68% were negative, and 12% were non-conclusive reactions. In cattle greater than 5 years of age, 5.5% were positive and 94.5% were negative. In total, 18.4% of the samples were positive, 71.3% were negative, and 10.3% were inconclusive (Figure-1a). In Mount Lebanon, 7.5% of the samples taken from cattle aged 2-5 years were positive and 92.5% were negative. On the other hand, all samples taken from cattle aged more than 5 years were negative. In total, 7% of the samples were positive and 93% were negative (Figure-1b).

### Bovine brucellosis rate by RBT

**Figure-2:**
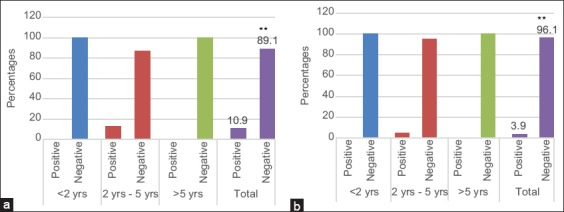
Variation of Bovine brucellosis rate by RBT with age in the two selected regions: Bekaa (a) Mount lebanon (b).

The results of the serological test in RBT showed that the rate of brucellosis in cattle varied with age in the two selected regions. In Bekaa and Mount Lebanon, all samples were taken from cattle aged <2 years old and over 5 years of age were negative reactions, while samples taken from cattle aged between 2 and 5 years were 13% positive and 87% negative in Bekaa. In total, there were 11% of positive and 89% of negative samples in Bekaa (Figure-2a). In Mount Lebanon, 4.8% of the samples taken from cattle aged 2-5 years were positive and 95% were negative. In total, there were 3.9% of positive and 96.1% of negative samples (Figure-2b).

### Bovine brucellosis rate by B. abortus indirect ELISA

**Figure-3:**
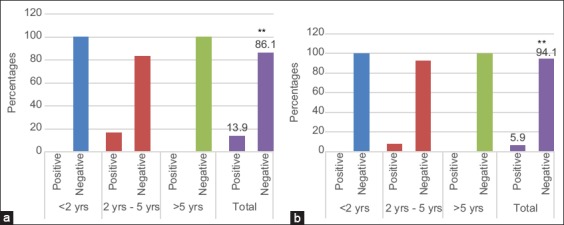
Variation of Bovine brucellosis rate by Elisa abortus indirect test with age in the two selected regions: Bekaa (a) Mount lebanon (b).

The results of the ELISA abortus indirect test showed that the rate of brucellosis in cattle varies with age in the two selected regions. In Bekaa, as in Mount Lebanon, all samples taken from cattle aged <2 years old and over 5 years of age were negative reactions. In Bekaa, the samples taken from cattle aged between 2 and 5 years were 16.5% positive and 83.4% negative. In Bekaa as well, the total percentage of positive samples was 13.9% positive and 86.1% negative (Figure-3a). In Mount Lebanon, the samples taken from cattle aged between 2 and 5 years were 7.26% positive and 92.4% negative. In total, there were 5.9% of positive and 94.1% of negative samples in Mount Lebanon (Figure-3b).

### Bovine brucellosis rate by competitive ELISA

Competitive ELISA was performed for the confirmation process. Based on the previous results, 27 positive samples and 85 negative samples were selected from Bekaa and used for this test. This test was performed on 73% (n=27) of the positive samples and 27% (n=85) of the negative samples. The results showed that milk test or MRT carried the highest percentage of positive samples with 18.4% in Bekaa and 6.9% in Mount Lebanon followed by the indirect ELISA test with 13.9% of positive samples in Bekaa and 5.8% in Mount Lebanon, then the RBT with 11% of positive samples in Bekaa and 4% in Mount Lebanon.

### Positive responses detected by rose Bengal after vaccination

**Figure-4:**
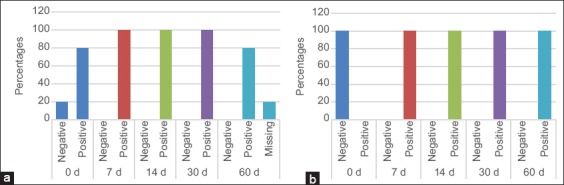
Results of Rose Bengal test performed on the different samples of group 1 (a) and group 2 (b).

Blood samples were taken from animals before and after vaccination. The results are shown in Figure-4a on the 1^st^ day before vaccination (day 0), 80% of cattle showed a negative response while 20% had a positive response, which could be interpreted as being a subclinical infection with brucellosis. About 100% of heifers had a negative response before vaccination (Figure-4b). These animals were not infected with the bacterium. The RBT yielded positive resulted after 7-day post-vaccination with the vaccine (full dose).

### Development of an immune response to brucellosis after vaccination

**Figure-5:**
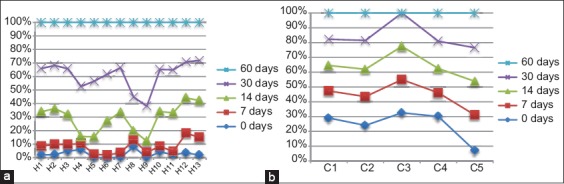
Results of iELISa test formed by individual animals at different days post vaccination for group 1 (a) and group 2 (b).

Figure-5a of Group 1 showed that all cows were the positive ones for the previous infection except cow 5 at the initial day. Seven days post-vaccination, the mature cows were faster to develop immunity against the pathogen with the help of a live attenuated vaccine regarding the strain used in this case. The observed results in the four animals previously positive before vaccination were different 7 days after vaccination, but this is irrelevant since the test is only qualitative, the immune response is, however, stable. On the study of serum samples taken on day 14 post-vaccination, the graph showed that Group 1 maintained immunity between 50% and 60%, denoting stability in the humoral immune response. These results will stay within their current range up until the end of the experiment on day 60.

Figure-5b of Group 2 shows that all individuals had same response on the 1^st^ day of sampling post-vaccination. About 100% of animals showed no positive results for the presence of antibodies. A result up to this date for Group 1 allowed us to postulate that immunity formation was slower in calves than in full-grown animals. On the 14^th^ day of sampling, the first results underlining immune response for brucellosis in heifers were the cases of numbers between 10% and 40%. Fourteen days post-vaccination, five remaining heifers H4, H5, H6, H8, and H9 were still unprotected against brucellosis infection. On day 30, only two heifers showed positive results in the serum test iELISA, while all other animals in the group were positive for *Brucella-* specific antigens. These results concluded that at 30-day post-vaccination, more than 85% of sexually immature animals would have elaborated a satisfactory immunity against brucellosis. On the last day of the follow-up (day 60), all heifers on the 2^nd^ group were positive for *Brucella-* specific antigens, which is the desired outcome of the vaccination program. This allows postulating that immunity in 100% of sexually immature animals is reached after a maximum of 60-day post-vaccination in this study. It is not possible to compare immunogenicity between vaccines. For these reasons, further tests were performed using PCR.

### Detection of PCR productsin electrophoresis gels

The aim of PCR test was to follow up on shedding or dissemination of the vaccinated strain in blood. The PCR yielded negative results for all the animals prior and post-vaccination (S1, S2, S3, and S4 are examples of samples). We concluded that the shedding and dissemination period of strain in vaccinated animals were <7 days. It means that *B. melitensis* vaccine is safe to use in cattle with low virulence of strain proven by <7 days shedding/dissemination period. In other words, the results regarding safety of the use of the strain in this vaccine were assessed using *B. melitensis*-specific conventional PCR test that showed negative results before and after vaccination. These results were either due to relatively low shedding and dissemination period of <7 days or could be related to a non-shedding or dissemination at all following vaccination. In both cases, the results were in favor of using the melitensis strain in cattle due to low virulence of the strain in this vaccination program.

**Figure-6:**
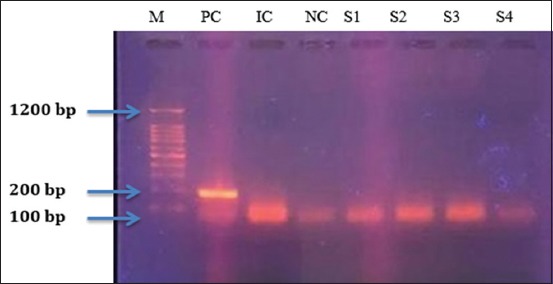
Polymerase chain reaction assay on gel agarose. Lane M: Brig TM molecular weight marker (Bioingentech Ltd.). Lane 1 PC: *Brucella melitensis* positive control, 185 bp. Lane 2 IC: Internal control, 140 pb. Lane 2 NC: Negative control. S1: Sample 1, S2: Sample 2, S3: Sample 3, S4: Sample 4.

The weight marker (M) was used to assess all parameters. Any sample with a molecular weight equal to the expected PCR product size was judged positive. Any fluorescent line (representing molecular weight) that was over or under that measure was considered a negative result. The results were studied individually per animal throughout the study and PCR yielded negative results for all the animals at day 0 (before vaccination), on 7-day post-vaccination and over the remaining days into the study. Both blood and milk samples were negative for the presence of the vaccine strain. This finding goes along those reported in Al-Majali *et al*. [11]. In this study, PCR test showed in Figure-6 that the shedding and dissemination period in vaccinated animals were <7 days or none with the vaccine, which demonstrated the safety of vaccine in cattle. Low virulence was proven by <7 days shedding/dissemination period (S1, S2, S3, and S4 are examples of negative results for the presence of the bacterium). After the evaluation of results using PCR, it can be said that vaccine is safe for use in cattle and in endemic regions where it is desired to protect animals and their productivity along with minimizing human exposure to the disease.

## Discussion

Based on studies made during the past years, brucellosis remains a serious endemic zoonotic disease in Lebanon and nearby countries. Our main research was to investigate the prevalence of brucellosis in different regions in Lebanon. Using a competitive ELISA test, in Bekaa and Mount Lebanon, the rate of brucellosis infection was 10% and 5.3%, respectively. The results of Bekaa valley showed an increase in the rate of *Brucella* infection by 4% in comparison to the study using the ELISA test during 2013 and 2017 (Dr. Daoud thesis). iELISA demonstrates high specificity and moderate sensitivity, whereas C-ELISA is highly specific and discriminative between antibodies from vaccination and those from infection [12-14].

In the majority of studies done, the seroprevalence of brucellosis was higher in females than in males as was seen by Adamu *et al*. [15] where females had higher seroprevalence than males by 13%. Females shed the disease more than males, especially due to abortions and all discharges, and they are present for a longer period of time in the herd as was explained in a study by Kanouté *et al*. [16]. Approximately, all the cattle tested were of Holstein breed. Some studies proved that the Holstein breed is more susceptible to brucellosis [17,18].

The persistence of the infection in the country leads to major economic losses as well as its toll on public health as can be seen in the general surveillance data conducted by the Ministry of Public Health every year. To confirm the previous statement, a study in 2015 [1] showed that the Middle East region still battles with the ongoing *Brucella* infection in different countries. While some countries have started vaccination programs for various ruminants, others are lacking any support neglecting the matter due to political reasons. One of the major thoughts of reason for the increased infection of brucellosis in the Bekaa region is the Syrian refugees. Syria reported high incidences of brucellosis and increased, especially during the Syrian war, where there was a lack of veterinary control [19]. While to this day, there still is deficient control and surveillance on the exchange and exporting of animals, i.e., the animal movements between Lebanon and nearby countries, the lack of monitoring and screening process of possible disease carriers before the introduction of these animals into a herd must be highlighted [20].

Other major risk factors affecting the widespread disease in Lebanon are the traditional consumption of raw unpasteurized dairy products, the improper handling of animals and its milk, and the contaminated environment where the animal is kept, while there are no appropriate hygienic standards used in milking machines as well as in case of the infected fecal matter and uterine secretions [21]. Indeed, the lowest human incidence in Lebanon was found in Beirut where little to no farms are present inside the city and it is assumed that its residents consume pasteurized and packaged dairy products.

The study on the vaccine showed the development of immunization against *Brucella* across 60 days in five lactating non-pregnant cows and 13 heifers where all animals developed a positive response to immunization. Results regarding the safety of the use of the strain in this vaccine were assessed using *B. melitensis*-specific conventional PCR test, which showed negative results before and after vaccination. These results were either due to relatively low shedding and dissemination period of <7 days or could be related to a non-shedding or dissemination at all following vaccination. In both cases, the results were in favor of using the melitensis Rev.1 strain in cattle due to low virulence of the strain in this vaccination program. This shows the possibility to use this vaccine in decreasing the chance of infection in herds. In case of mass eradication, broader research must be established to show the full efficacy of this vaccine.

In Lebanon, the lack of consistent research to little feedback on occurring illness or symptoms creates a huge hole in aiding to control the spread of the disease. As a start, sustainable surveillance and monitoring programs in addition to a vaccination protocol must be managed in several farms, especially in places where pasteurization is avoided. To control this infection, several protocols must be undertaken from educating the consumer and the farmer on the dangers of brucellosis and on the raw products as a route of infection, to vaccinations and quarantine of the infected animal. The latter is a bit overshadowed by the financial losses. Farmers and personnel should abide to Hazard Analysis and Critical Control Point practices to minimize the risk of infections that can be transmitted through direct contact, especially that the overall food safety infrastructure in Lebanon needs significant improvements [21-23]. In addition, necessary precautions need to be considered in case of transportation or introducing a new animal to the farm. Necessary measures must take place in the nearest time since some strains of *Brucella* species are showing resistance to several antimicrobials drugs as seen in a 2010 study [24].

## Conclusion

Brucellosis is a zoonosis of bacterial origin, highly contagious and causes significant economic losses in Lebanon. Health and medical prevention, one of which is vaccination, help control the disease and reduce economic losses only in animals due to the high risk of infection in humans. Our results showed that the highest rate of *Brucellae* was found in the Bekaa region with correlation with the epidemiological study in human cases. After vaccination of cows and heifers at different times, the results showed that all the vaccinated animals have developed an immune response to brucellosis 60 days after vaccination. This vaccine can be considered as a good, stable, and preventative vaccine to protect against brucellosis in animals and can indirectly protect public health from infection. More complete and accurate documentation could be done with studies regarding the melitensis vaccines and using a bigger sample size in future studies. The results could show more hidden aspects of immunogenicity and safety issues regarding this vaccine; for example, vaccination of pregnant animals in different stages of their pregnancy, or vaccinating males, whether sexually mature or immature.

## Authors’ Contributions

HH and NN wrote the manuscript; AS carried out the statistical analysis and helped in writing the manuscript; RAH collected the samples and helped in the laboratory work; JE helped in the laboratory work; RA designed the study and did the laboratory analysis. HH and AS contributed equally to the work. All authors read and approved the final manuscript.
